# Factors associated with outcome after successful radiological intervention in arteriovenous fistulas: A retrospective cohort

**DOI:** 10.1177/1129729819845991

**Published:** 2019-05-14

**Authors:** Sotiria Manou-Stathopoulou, Emily J Robinson, John Julian Harvey, Narayan Karunanithy, Francis Calder, Michael G Robson

**Affiliations:** 1 Renal, Transplant and Urology Directorate, Guy’s Hospital, Guy’s and St Thomas’ NHS Foundation Trust, London, UK; 2 Department of Biostatistics & Health Informatics, King’s College London, London, UK; 3 Department of Interventional Radiology, Guy’s Hospital, Guy’s and St Thomas’ NHS Foundation Trust, London, UK; 4 School of Immunology and Microbial Sciences, King’s College London, London, UK; 5 MRC Centre for Transplantation, King’s College London, London, UK

**Keywords:** Access, angioplasty, dialysis, fistula, balloon, vascular

## Abstract

**Introduction:**

Arteriovenous fistulas are the best form of vascular access for haemodialysis. A radiological balloon angioplasty is the standard treatment for a clinically relevant stenosis, but the recurrence rate is high. Data on factors associated with recurrence are limited.

**Methods:**

A single centre, retrospective analysis was performed for 124 consecutive patients who had successful interventions for dysfunctional arteriovenous fistulae, to examine factors associated with post-intervention patency. Follow-up was at least 1 year for all patients. Variables associated with primary and cumulative patency were pre-specified and assessed using both un-adjusted (univariate) and adjusted Cox proportional hazards models. Analysis was repeated for a subgroup of 80 patients with a single lesion only in order to examine the potential effects of stenotic lesion characteristics on patency.

**Results:**

Factors found to have a significant association with poorer outcomes (less time to loss of patency) included thrombosis at the time of intervention and a history of previous intervention. Fistula age (log days) was significantly associated with better outcomes (greater time to loss of patency). Non-white ethnicity, lesion length, and patient age were also significantly associated with accelerated loss of patency.

**Discussion:**

The factors we have identified as linked to poor outcome may help to identify patients in whom a balloon angioplasty is unlikely to provide a durable outcome. This may prompt exploring alternative treatment or dialysis options at an early stage.

## Introduction

The initial therapy for a clinically relevant stenosis in an arteriovenous fistula (AVF) is balloon angioplasty. A major concern is efficacy and longevity of the result after the treatment. Turmel-Rodrigues et al.^1^ reported the outcomes of interventional salvage of dysfunctional and thrombosed haemodialysis circuits. There were 220 cases in the dysfunctional AVF group. The 6-, 12- and 24-month primary patency (AVF working with no repeat intervention) reported were 67%, 51% and 37% for forearm AVF, and 57%, 35% and 24% for upper arm AVF, respectively. More recently, Bountouris et al.^2^ reported the outcomes after 159 percutaneous transluminal angioplasties (PTAs) in AVFs. Post-intervention primary patency (PIPP) at 6, 12 and 24 months was 61%, 42% and 35%, respectively. Primary assisted patency (AVF working regardless of repeat intervention) was 89% and 85% at 6 and 12 months, respectively. Although there have been some exceptions,^3,4^ most other studies have reported similar primary patency rates of around 40%–50% at 1 year.^5–7^

There are limited data available regarding clinical factors predicting outcome after balloon angioplasty. Although the majority of potential factors are not modifiable, it remains important to understand how they affect outcome. If the outcome is unlikely to be successful, then the possibility of surgical revision or new access should be considered. These options all come with cost, inconvenience and discomfort. An estimate of the expected outcome of a balloon angioplasty is therefore important information in determining the best course of action.

In this report, we describe a single centre experience of balloon angioplasty in 124 consecutive patients. In order to assess the effect of lesion anatomy, we also performed a second analysis in the subgroup of 80 patients with a single lesion only.

## Methods

### Patient population

We undertook a retrospective analysis of consecutive cases referred to interventional radiology, over an 18-month period between April 2013 and October 2014, with a dysfunctional AVF at our institution. We included for analysis the patients who had a technically successful interventional procedure: <30% residual stenosis in the access circuit. AVFs that had not been used for dialysis and AVFs thrombosed at the time of intervention were included. Balloon angioplasties were performed as follows. Prior to treatment, 3000–5000 IU of heparin was administered. A Bard Conquest or Dorado high-pressure balloon was used as standard and was sized to the nominal vein diameter. It was inflated to ensure obliteration of the lesion waist with a minimum duration of balloon inflation of 1 min. If the radiological result was suboptimal, further prolonged inflations of up to 5 min were performed and/or balloon diameter was upsized by 1–2 mm. Drug-coated balloons and cutting balloons were not routinely used. Thrombosed AVFs were treated with pharmacomechanical thrombectomy with the Angiojet^™^ (Boston Scientific) device followed by balloon angioplasty of any significant stenosis. Each patient was considered only once, using the first intervention they underwent in the study period. The end of follow-up was 1 year after the last procedure; therefore, all patients were followed up for at least 1 year. Tests were repeated twice for the two groups of patients: (1) all patients with one or more lesions (N = 124) and (2) subgroup of patients with one lesion only (N = 80). Data were obtained from a retrospective review of electronic patient records.

### Definitions

Standardised definitions were used.^8^ PIPP ended when any of the following occurred: (a) access circuit thrombosis, (b) an intervention (either radiological or surgical) anywhere in the access circuit, or (c) the access circuit was abandoned due to an inability to treat any lesion. Post-intervention cumulative patency (PICP) was considered to end when the AVF was abandoned, regardless of radiological or surgical intervention, with or without a thrombosis event. Censoring occurred when the patient died or had a transplant before reaching the outcome(s). Both outcomes were calculated as time (days) between date of procedure and (a) end of patency, if the patient lost patency before end of follow-up; or (b) date of censor, if the patient had not lost patency before death or transplant; or (c) end of follow-up, if the patient has not lost patency before follow-up period. Variables to be assessed were identified a priori and all variables assessed are included in this report. In patients with one lesion only, the stenosis is classified as anastomotic if both vein and artery were affected, and juxtaanastamotic if they were within 3 cm of the anastomosis with the artery not affected. Central stenosis was defined as being central to the thoracic inlet.

### Statistics

**Figure 1.**
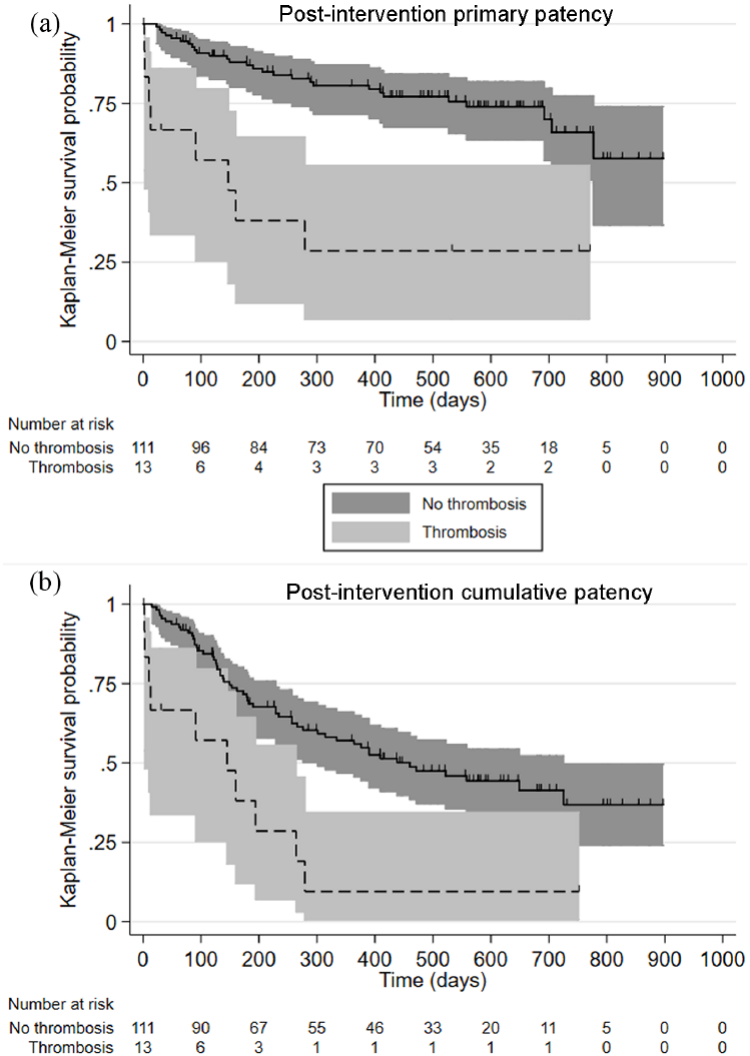
(a) Kaplan–Meier survival function of time to post-intervention primary patency for patients with thrombosis compared to those without thrombosis, using the total sample of patients (one or more lesions). (b) Kaplan–Meier survival function of time to post-intervention cumulative patency for patients with thrombosis compared to those without thrombosis, using the total sample of patients (one or more lesions). Log-rank tests were statistically significant (p < 0.001) for both comparisons.

Descriptive statistics have been presented in n (%), mean (SD) or median (interquartile range (IQR)), as appropriate. For the inferential analysis, first, un-adjusted Cox proportional hazard models were fitted separately for each of the patient characteristics in Table 1, to test which (if any) of the variables were univariately associated with either of the two outcomes within the two patient groups. Second, all relevant patient characteristics were then fitted into one adjusted model, per outcome, per group, to test whether any of the patient characteristics were significantly associated with the two outcomes when controlling for all other variables. Finally, stepwise estimation was performed using backward-selection, using α = 0.05 for removal from the Cox proportional hazards model: all relevant variables were included in an adjusted model and removed one by one if p < 0.05, leaving only significant covariates in the model. Graphical methods such as Kaplan–Meier survival curves were used to assess violations of the proportional hazards assumption, as well as Schoenfeld residuals. Log-rank tests were also used to assess equality of survival functions for group variables in Figure 1.

**Table 1.** Descriptive statistics of all patient variables regarded as potential predictors in the analysis.

Variable	All patients (N = 124)	Patients with one lesion only (N = 80)
Age (years)		
Mean (SD) [range]	62.6 (15.1) [23, 90]	63.8 (16.0) [23, 90]
Age n (%)		
<65 years	61 (49.2)	36 (45.0)
⩾65 years	63 (50.8)	44 (55.0)
Sex n (%)		
Male	59 (47.6)	38 (47.5)
Female	65 (52.4)	42 (52.5)
Ethnicity n (%)		
Black	45 (36.3)	28 (35.0)
Asian	12 (9.7)	7 (8.7)
White	57 (46.0)	41 (51.3)
Other	9 (7.3)	4 (5.0)
Missing	1 (0.8)	–
Age of fistula (days)		
Median (IQR) [range]	477.5 (211.5, 1243.5) [49, 4570]	472 (188, 1321) [49, 4570]
Statin therapy intensity^†^ n (%)		
None	60 (48.4)	37 (46.3)
Low	5 (4.0)	4 (5.0)
Medium	40 (32.3)	30 (37.5)
High	19 (15.3)	9 (11.2)
History of coronary artery disease (CAD) n (%)		
Yes	25 (20.2)	14 (17.5)
No	99 (79.8)	66 (82.5)
History of peripheral vascular disease (PVD) n (%)		
Yes	14 (11.3)	10 (12.5)
No	110 (88.7)	70 (87.5)
Anticoagulation n (%)		
Yes	7 (5.6)	3 (3.8)
No	117 (94.4)	77 (96.2)
Diabetes n (%)		
Yes	52 (41.9)	29 (36.3)
No	72 (58.1)	51 (63.7)
Type of access n (%)		
Brachiobasilic	43 (34.7)	28 (35.0)
Brachiocephalic	70 (56.4)	44 (55.0)
Radiocephalic	11 (8.9)	8 (10.0)
Lesion length (mm) Mean (SD) [range]	NA	(n = 76) 3.8 (1.8) [1, 12]
Number of lesions n (%)		
1	80 (64.5)	
2	41 (33.1)	
3	2 (1.6)	
4	1 (0.8)	NA
Lesion site n (%)		
Anastomotic		8 (10.0)
Perianastamotic		14 (17.5)
Mid-limb		27 (33.8)
Swing point/cephalic arch		25 (31.2)
Central stenosis		4 (5.0)
Arterial	NA	2 (2.5)
Thrombosis n (%)		
Yes	13 (10.5)	7 (8.7)
No	111 (89.5)	73 (91.3)
Previous interventions n (%)		
None	82 (66.1)	54 (67.5)
1	30 (24.2)	19 (23.8)
2 or more	12 (9.7)	7 (8.7)

IQR: interquartile range.

Low: Fluvastatin (20-–40 mg), Lovastatin (20 mg), Simvastatin (10 mg) or Pravastatin (10–20 mg); medium: Atorvastatin (10–20 mg), Fluvastatin (80 mg), Pravastatin (40–80 mg), Rosuvastatin (5–10 mg) or Simvastatin (20–40 mg); high: Atorvastatin (40–80 mg) or Rosuvastatin (20–40 mg).

† None: no statin therapy.

Lesion site and lesion length were excluded from the first set of tests when including all patients (with one or more lesions) as they would be measurements of one lesion only; similarly, number of lesions was not applicable as a factor in the second set of tests when removing patients with multiple lesions. For some variables, multiple categories were broadened or merged in order to create binary variables. This was due to small numbers in sub-categories or to aid interpretation. Variables that were binarised were age (<65 years vs ⩾65 years), ethnicity (white vs non-white), statin therapy (none vs any), number of lesions (1 vs 2 or more), and previous intervention (0 vs 1 or more). Age of fistula was log-transformed to deal with the positive skewness of the variable, as well as the non-linear relationship between age of fistula and the two outcomes. Software used was Stata version 15.0 (StataCorp, Texas).

## Results

Patient variables that we considered potentially associated with the outcomes are described in Table 1, and all characteristics appear to be similar between all patients (N = 124) and those with a single lesion only (N = 80). Table 2 shows descriptive data for the two patency outcomes and the censor variables. Out of all 124 patients, 6- and 12-month PIPP loss was 32.3% and 44.4%, respectively. PICP loss for the 124 patients at 6 and 12 months was 16.9% and 22.6%, respectively. For the 80 patients with a single lesion, PIPP loss at 6 and 12 months was 31.3% and 43.8%, respectively. PICP loss for the 80 patients at 6 and 12 months was 13.8% and 20.0%, respectively.

**Table 2.** Descriptive statistics of all outcome or censored variables by patency loss.

Variable	All patients N = 124		Patients with one lesion only N = 80	
	PI primary patency loss	PI cumulative patency loss	PI primary patency loss	PI cumulative patency loss
Censored within follow-up n (%)				
Total	21 (16.9)	27 (21.8)	14 (17.5)	17 (21.3)
Death	10 (8.1)	14 (11.3)	9 (11.3)	12 (15.0)
Transplant	11 (8.9)	13 (10.5)	5 (6.2)	5 (6.3)
Overall loss of patency n (%)				
Yes	66 (53.2)	36 (29.0)	40 (50.0)	20 (25.0)
No	58 (46.8)	88 (71.0)	40 (50.0)	60 (75.0)
Loss of patency at… n (%)				
6 months	40 (32.3)	21 (16.9)	25 (31.3)	11 (13.8)
12 months	55 (44.4)	28 (22.6)	35 (43.8)	16 (20.0)
Time (days) to patency loss	(n = 66)	(n = 36)	(n = 40)	(n = 20)
Median (IQR) [range]	151 (89, 276) [2, 725]	148 (57, 291) [2, 777]	138.5 (89, 260.5) [10, 725]	136 (57, 291) [10, 558]

IQR: interquartile range.

The number of patients who were censored for death or transplant, and the number of patients who lost post-intervention (PI) primary patency and/or PI cumulative patency within the follow-up period.

Results of the Cox proportional hazards analyses for all 124 patients are shown in Table 3, reported as hazard ratios (HR) and 95% confidence intervals (CI). Thrombosis at the time of presentation and younger fistulas (log days) were significantly associated with worse outcomes (PIPP and PICP) in all three tests (adjusted and un-adjusted). Kaplan–Meier plots showing the effect of thrombosis on PIPP and PICP are shown in Figure 1. Previous intervention and non-white ethnicity were also associated with both outcomes in the adjusted and stepwise models. Age of patient remained significant in the adjusted and stepwise models for time to PICP.

**Table 3.** Results of (a) un-adjusted Cox proportional hazards models to test univariate associations between patient characteristics and time to end (days) post-intervention (PI) patency loss of PI cumulative patency loss; (b) adjusted Cox proportional hazards model controlling for all patient characteristics; and (c) stepwise estimation, for all patients (with one or more lesions) (N = 124).

Patient characteristics; potential predictors of the outcome(s)	(a) Un-adjusted Cox proportional hazards models HR (95% CI)		(b) Adjusted Cox proportional hazards model HR (95% CI)		(c) Stepwise selection Sig. or NS	
	PI primary patency loss	PI cumulative patency loss	PI primary patency loss	PI cumulative patency loss	PI primary patency loss	PI cumulative patency loss
Age						
<65 years (ref)	–	–	–	–		
⩾65 years	0.92 (0.57, 1.50)	1.38 (0.71, 2.66)	1.20 (0.66, 2.18)	*2.31 (1.05, 5.08)	NS	*Sig.
Sex						
Female (ref)	–	–	–	–		
Male	1.17 (0.72, 1.90)	1.25 (0.65, 2.41)	1.10 (0.64, 1.90)	1.02 (0.48, 2.16)	NS	NS
Ethnicity						
Non-white (ref)	–	–	–	–		
White	0.66 (0.40, 1.08)	0.63 (0.32, 1.24)	*0.46 (0.26, 0.83)	*0.42 (0.18, 0.99)	*Sig.	*Sig.
Age of fistula (log days)	*0.80 (0.65, 0.99)	*0.72 (0.53, 0.97)	*0.67 (0.51, 0.88)	*0.53 (0.35, 0.79)	*Sig.	*Sig.
Statins						
None (ref)	–	–	–	–		
Any	1.63 (1.00, 2.68)	1.76 (0.89, 3.47)	1.53 (0.80, 2.93)	0.88 (0.36, 2.15)	NS	NS
CAD						
No (ref)	–	–	–	–		
Yes	0.94 (0.52, 1.70)	1.25 (0.59, 2.66)	0.68 (0.32, 1.43)	1.11 (0.42, 2.89)	NS	NS
PVD						
No (ref)	–	–	–	–		
Yes	1.56 (0.77, 3.16)	1.83 (0.76, 4.43)	1.61 (0.70, 3.71)	1.09 (0.37, 3.18)	NS	NS
Anticoagulation						
No (ref)	–	–	–	–		
Yes	1.49 (0.60, 3.73)	1.41 (0.43, 4.62)	2.24 (0.79, 6.35)	2.30 (0.59, 9.00)	NS	NS
Diabetes	–					
No (ref)	–	–	–	–		
Yes	1.18 (0.72, 1.92)	1.80 (0.94, 3.48)	0.86 (0.46, 1.62)	1.55 (0.61, 3.94)	NS	NS
Type of access						
Brachiobasilic (ref)	–	–	–	–	–	–
Brachiocephalic	0.93 (0.56, 1.56)	1.04 (0.52, 2.09)	1.05 (0.60, 1.85)	1.09 (0.49, 2.44)		
Radiocephalic	1.13 (0.46, 2.77)	1.00 (0.28, 3.53)	1.46 (0.55, 3.85)	1.33 (0.33, 5.35)	NS	NS
Number of lesions						
1 (ref)	–	–	–	–		
2+	1.19 (0.73, 1.95)	1.49 (0.77, 2.88)	1.20 (0.69, 2.08)	1.33 (0.63, 2.78)	NS	NS
Thrombosis						
No (ref)	–	–	–	–		
Yes	**3.14 (1.59, 6.20)	**4.68 (2.11, 10.36)	**5.25 (2.27, 12.12)	**13.34 (4.43, 40.18)	**Sig.	**Sig.
Previous intervention						
0 (ref)	–	–	–	–		
1+	1.09 (0.65, 1.81)	0.95 (0.47, 1.91)	*2.12 (1.10, 4.06)	*2.90 (1.18, 7.09)	*Sig.	*Sig.

HR: hazard ratio; CI: confidence interval; ref: reference category; Sig.: significant (p < 0.05) in stepwise regression; NS: not significant in stepwise regression and therefore removed from model; CAD: coronary artery disease; PVD: peripheral vascular disease.

* Statistically significant at the p < 0.05 level; **statistically significant at the p < **0.001 level**.

We performed a second analysis for patients with a single lesion because it was not possible to assess the effect of lesion location and length if there were multiple lesions. It was impossible to study, for example, the effect of having a lesion at the anastomosis if there was an anastamotic lesion present, and a second lesion present in the venous segment and/or cephalic arch. Results of analyses for the 80 patients with a single lesion only is shown in Table 4. Younger fistulas (log days) were significantly associated with both outcomes in the adjusted model. Statin use was associated with a shorter time to PICP in the unadjusted and stepwise models. Lesion length was significantly associated with time to PICP in the unadjusted, adjusted and stepwise models.

**Table 4.** Results of (a) un-adjusted Cox proportional hazards models to test univariate associations between patient characteristics and time to end (days) of post-intervention (PI) patency loss of PI cumulative patency loss, (b) adjusted Cox proportional hazards model controlling for all patient characteristics, and (c) stepwise estimation, for patients with one lesion only (N = 80).

Patient characteristics; potential predictors of the outcome(s)	(a) Un-adjusted Cox proportional hazards models HR (95% CI)		(b) Adjusted Cox proportional hazards model HR (95% CI)		(c) Stepwise selection Sig. or NS	
	PI primary patency loss	PI cumulative patency loss	PI primary patency loss	PI cumulative patency loss	PI primary patency loss	PI cumulative patency loss
Age						
<65 (ref)	–	–	–	–		
⩾65	0.79 (0.43, 1.47)	1.34 (0.55, 3.28)	0.75 (0.29, 1.89)	1.15 (0.27, 4.83)	NS	NS
Sex						
Female (ref)	–	–	–	–		
Male	1.19 (0.64, 2.22)	1.28 (0.53, 3.10)	1.84 (0.83, 4.06)	2.76 (0.70, 10.89)	NS	NS
Ethnicity						
Non-white (ref)	–	–	–	–		
White	0.73 (0.39, 1.37)	0.82 (0.34, 1.97)	0.60 (0.26, 1.39)	1.29 (0.35, 4.65)	NS	NS
Age of fistula (log days)	0.84 (0.66, 1.09)	0.74 (0.52, 1.06)	*0.65 (0.44, 0.97)	*0.48 (0.23, 0.99)	NS	NS
Statins						
None (ref)	–	–	–	–		
Any	1.59 (0.84, 2.99)	*3.10 (1.13, 8.54)	2.34 (0.99, 5.53)	3.47 (0.73, 16.49)	NS	*Sig.
CAD						
No (ref)	–	–	–	–		
Yes	0.59 (0.23, 1.51)	1.25 (0.42, 3.75)	0.76 (0.22, 2.66)	5.49 (0.92, 32.64)	NS	NS
PVD						
No (ref)	–	–	–	–		
Yes	1.19 (0.47, 3.05)	1.45 (0.42, 4.97)	0.62 (0.12, 3.07)	0.67 (0.06, 7.91)	NS	NS
Anticoagulation						
No (ref)	–	–	–	–		
Yes	1.45 (0.35, 6.06)	1.46 (0.20, 11.00)	4.87 (0.31, 76.75)	NA	NS	NS
Diabetes						
No (ref)	–	–	–	–		
Yes	1.04 (0.54, 2.02)	1.80 (0.74, 4.36)	1.29 (0.51, 3.26)	2.12 (0.45, 10.10)	NS	NS
Type of access						
Brachiobasilic (ref)	–	–	–	–		
Brachiocephalic	0.72 (0.37, 1.39)	0.66 (0.26, 1.67)	0.79 (0.32, 1.97)	0.49 (0.12, 1.92)		
Radiocephalic	0.92 (0.31, 2.75)	0.73 (0.16, 3.37)	1.15 (0.28, 4.70)	0.89 (0.09, 8.64)	NS	NS
Lesion length (mm)	1.06 (0.90, 1.24)	*1.21 (1.02, 1.43)	1.06 (0.81, 1.39)	*1.89 (1.26, 2.84)	NS	*Sig.
Lesion site						
Anastomotic (ref)	–	–	–	–		
Perianast	1.01 (0.23, 4.55)	0.32 (0.03, 3.50)	3.37(0.43, 26.63)	0.29 (0.01, 8.21)		
Mid-limb	1.50 (0.43, 5.18	0.89 (0.19, 4.31)	3.22 (0.54, 19.08)	0.34 (0.02, 4.71)		
Swing point	1.42 (0.41, 4.97)	1.25 (0.27, 5.79)	3.85 (0.74, 20.08)	2.36 (0.25, 22.36)		
Central stenosis	1.82 (0.37, 9.11)	0.91 (0.08, 10.03)	10.44 (1.18, 92.51)	4.78 (0.18, 125.04)		
Arterial	0.69 (0.07, 6.80)	NA	NA	NA	NS	NS
Thrombosis						
No (ref)	–	–	–	–		
Yes	1.96 (0.77, 5.03)	2.42 (0.71, 8.27)	2.32 (0.64, 8.42)	3.54 (0.55, 22.93)	NS	NS
Previous intervention						
0 (ref)	–	–	–	–		
1+	1.06 (0.55, 2.05)	−0.81 (0.31, 2.10)	1.29 (0.46, 3.61)	0.35 (0.06, 1.94)	NS	NS

HR: hazard ratio; CI: confidence interval; ref: reference category; Sig.: significant (p < 0.05) in stepwise regression; NS: not significant in stepwise regression and therefore removed from model; NA: not applicable due to small n; CAD: coronary artery disease; PVD: peripheral vascular disease.

* Statistically significant at the p < 0.05 level; **statistically significant at the p < 0.001 level.

## Discussion

In this study, we have found a significant association between poorer outcomes (less time to loss of patency) and thrombosis at the time of intervention or a history of previous intervention. Fistula age (log days) was significantly associated with better outcomes (greater time to loss of patency). Non-white ethnicity, lesion length, and patient age were also significantly associated with accelerated loss of patency.

A publication in 2014 provides a systematic review of previous research.^9^ The authors identified 10 studies, which examined factors affecting primary patency after radiological intervention.^5–7,10–15^ We have included two additional papers published since this review in our discussion.^4,16^ Our overall patency rates (Table 2) are consistent with previous reports. These studies have a number of limitations. Follow-up is variable and in some cases short. Six studies did not use multivariable methods in their analysis. No study was explicit about missing data. Most studies did not comment on whether previous interventions had been performed. In some cases, the variables to be assessed were not pre-specified, and variables found to be non-significant were not reported. Patients were sometimes included more than once in the study if they had a second fistula or intervention in the same fistula. Most studies were retrospective.

These previous studies have had a similar definition of PIPP. We considered that either surgical and radiological endovascular intervention led to loss of primary patency, in keeping with standardised definitions.^8^ Our definition of PICP was not used in most. Instead, definitions such as ‘assisted primary patency’ (time to thrombosis or surgical intervention) and ‘secondary patency’ (time to surgical intervention or abandoning of the AVF) were used. We consider that the endpoint of PICP is more clinically relevant than these. In our report, surgical revision (with the same AVF preserved) is compatible with continued cumulative patency, in keeping with standardised definitions.^8^ From the perspective of both the patient and the clinical team, the important issue is how long their fistula lasts. A longer lasting fistula will also lead to reduced health care costs. If a fistula fails, then alternative and inferior access such as a central venous catheter or a synthetic graft, associated with higher morbidity, may be needed.

We found an effect of both previous intervention and thrombosis on both primary and cumulative patency in the total group of patients. It is not clear if the outcome would have been better if the thrombosis had been prevented. However, neither variable was significantly associated with outcome when restricting the cohort to patients with a single lesion only. We do not know if this reflects the smaller sample size or a true difference in the effect of previous intervention and thrombosis in patients with a single lesion. Thrombosis was assessed as a variable affecting primary patency in only two studies,^6,12^ with neither showing an effect.

We found that an older fistula had an improved primary and cumulative patency. Three of seven studies found that newer fistulas had a shorter primary patency,^7,12,14^ which is in keeping with this report. This reflects the fact that older fistulas are more mature. We did not collect data on how many of the fistulas had been used for dialysis prior to intervention. It is possible that a worse outcome would have been seen in fistulas that had not been used. Some of our patients had not yet started haemodialysis so a lack of use for dialysis would not necessarily mean the fistula was not mature and suitable. It is not possible to comment on suitability for dialysis in a retrospective study, and this lack of assessment of fistula maturity is a limitation of our study.

We showed worse outcomes for non-white patients and for patients over 65 using both the adjusted and the step-wise model. Ethnicity and age were not assessed in most previous studies. Overall, our data for other baseline clinical and demographic variables are in keeping with previous studies, with the novel observation that ethnicity and age are relevant factors.

We found no effect for diabetes, previous peripheral vascular or coronary disease. Diabetes was associated with worse PIPP in two previous studies^4,10^ but not in eight others. Two studies looked at peripheral vascular disease and coronary artery disease, and found no effect for either. None of the previous studies found an effect of the number of lesions, also in keeping with our data. Previous studies have not reported the effect of previous intervention on primary patency.

We found that longer lesions had a reduced cumulative patency. Interpretation of previous published data on both lesion length and location is problematic. The current study is the only report we are aware of that describes the effect of lesion anatomy on outcome in patients with a single lesion. It is not clear how the presence of multiple lesions was accounted for and all series included a proportion of patients with more than one lesion. This may have obscured possible effects. Three studies found that longer lesions had a reduced PIPP,^3,12,16^ though lesion length had no effect in five other studies. One of these studies^16^ reported an effect of lesion location, suggesting that venous outflow predicted a worse outcome. In this study, 26% of patients had multiple lesions. Venous outflow lesions were stated as being present in 83%, but these were not subdivided into venous segment and cephalic arch making the findings difficult to interpret. Furthermore, only an unadjusted analysis was performed. Six other studies analysed the effect of lesion location with various different groups compared and no differences were found. In order to avoid the difficulties of interpreting the effect of lesion length and location with multiple lesions, we only assessed variables relating to lesion anatomy in 80 patients with a single lesion.

Our study is limited in that it is retrospective. However, it is one of the largest series reported, each patient is included only once, follow-up for all patients is at least 1 year, variables of interest were specified prior to the analysis, and we have used multivariable methods in our analysis, allowing us to relate several risk factors, considered simultaneously, to patency survival time. Our data differ from prior reports in a number of ways. First, we have repeated our analysis in patients with a single lesion only to include the effect of lesion anatomy. Second, we have analysed the effect of ethnicity and previous intervention and found effects for both. The current data may inform decisions about repeat intervention and the timing of surgery for new vascular access. In our experience, a young fistula, with previous interventions, and thrombosis at the time of presentation are a fistula that will not respond well to radiological intervention, in terms of patency outcomes. These findings may have been predicted by those with clinical experience. However, it is useful to have objective data to support these clinical impressions. In these patients, it may be wise to make plans for further access at the time of intervention or to consider not intervening at all. Further prospective data may soon be available from clinical trials assessing the effect of drug-coated balloons on balloon angioplasty, for example, the PAVE trial.^17^ In addition to assessing the study treatment, these trials will provide the opportunity to prospectively assess the effect of clinical variables on outcomes.
